# Disorders of Redox Homeostasis and Its Importance in Acrolein Toxicity

**DOI:** 10.3390/ijms26189047

**Published:** 2025-09-17

**Authors:** Magdalena Kwolek-Mirek, Roman Maslanka, Sabina Bednarska, Joanna Szczypek, Justyna Baran, Michał Przywara, Agnieszka Janeczko, Renata Zadrag-Tecza

**Affiliations:** Faculty of Biology, Nature Protection, and Sustainable Development, University of Rzeszów, 35-601 Rzeszów, Poland; rmaslanka@ur.edu.pl (R.M.); sbednarska@ur.edu.pl (S.B.); retecza@ur.edu.pl (R.Z.-T.)

**Keywords:** glucose-6-phosphate dehydrogenase, 6-phosphogluconate dehydrogenase, NADPH generation, NADPH/NADP^+^ ratio, GSH/GSSG ratio, ROS, redox homeostasis, acrolein, allyl alcohol, yeast

## Abstract

The maintenance of intracellular redox homeostasis depends on the GSH/GSSG pair, which is the primary intracellular redox buffer. However, the NADPH/NADP^+^ pair also plays a vital role in this process. The primary source of NADPH is the pentose phosphate pathway and deficiency in the enzymes responsible for NADPH production in this pathway leads to developing of alternative NADPH supply strategies. The choice of compensation strategy has several consequences for cells physiology. The present study investigates how *Saccharomyces cerevisiae* yeast strains defective in generating NADPH via the pentose phosphate pathway due to deletion of *ZWF1*, *GND1*, or *GND2* genes, respond to redox homeostasis disruption caused by allyl alcohol, a metabolic precursor of acrolein. Acrolein is a highly reactive aldehyde that rapidly depletes glutathione and triggers oxidative stress. Therefore, cells respond to acrolein through attempts to increase glutathione synthesis, but also by increasing NADPH production. The response requires coordinated action of glutathione- and NADPH-dependent systems. The high sensitivity of the Δ*gnd1* strain, which is unable to activate an adequate stress response, is evidence of this. The strategy employed by this strain to maintain redox homeostasis is inadequate and may even exacerbate allyl alcohol toxicity.

## 1. Introduction

Redox (reduction-oxidation) is a pivotal mechanism in living systems, whereby electrons are transferred from a donor (a reducing agent) to an acceptor (an oxidizing agent). Oxygen is the most prevalent oxidiser, although nitrogen and sulphur also participate in redox reactions [1]. Cellular redox homeostasis is a balance between reduction and oxidation reactions, and its maintenance depends on enzymes, small antioxidants, and reactive species [1,2]. Reducing force of the cells is provided by: the glutathione system, i.e., the reduced GSH in general and the intracellular GSH/GSSG redox potential; the thioredoxin system consisting of thioredoxins and thioredoxin reductases; and the redox-active cofactors, both the reduced NADPH and the NADPH/NADP^+^ ratio [2,3]. Redox homeostasis is essential for many cellular processes, including responses to reactive oxygen species (ROS), signaling, protection of protein thiols, as well as xenobiotics removal [4]. Living organisms can adapt to some extent to an altered redox homeostasis, for example, by upregulating the antioxidative defence (adaptive homeostasis). It is related to increasing the production of low molecular weight antioxidants and changing the expression and/or activity of antioxidant enzymes [1,3]. When these treatments are effective, redox homeostasis is restored, and the cell adapts to new conditions. However, in cases where an imbalance is severe or an imbalance-generated condition persists in the cell for an extended period, a cascade of cellular disruptions and damage can occur. Damage to cell components/macromolecules at the molecular level can result in damage to cellular organelles. This, in turn, can lead to cellular dysfunction or even cell death.

Acrolein (Acr) is a highly reactive α,β-unsaturated aldehyde commonly occurring in the environment. It is formed during the incomplete combustion of petrol, coal, wood, and plastic materials. It is also constituent of tobacco smoke. Acrolein can also be formed endogenously as a product of lipid peroxidation [5]. Acrolein is known for its potent electrophilic properties and rapid reaction with cellular soft nucleophiles, such as glutathione thiol groups and protein cysteine thiols [6,7]. Therefore, acrolein is a compound with a strong oxidising and redox homeostasis-disrupting effect observable in diverse cell types [8], including yeast *Saccharomyces cerevisiae* [9,10,11,12,13,14,15]. Glutathione binds with acrolein, leading to the formation of acrolein–GSH conjugates, which can occur either non-enzymatically or through glutathione S-transferases (GST) [5]. The main reactivity of acrolein with GSH and the subsequent disruption of cellular redox homeostasis led to increased ROS production in cells. These lead to: (i) massive damages to macromolecules, such as protein carbonylation, and DNA condensation and fragmentation; (ii) damages to cellular organelles/structures resulting, among others, in decreased actin microfilament dynamics, mitochondrial disintegration, and phosphatidylserine exposure on the cell surface; (iii) growth inhibition/arrest, as well as both apoptotic and necrotic cell death [9,10,11,13,14]. Moreover, the redox state of protein sulfhydryl groups is subject to dynamic changes due to unanticipated oxidative modification. Therefore, to maintain the redox balance, cells possess enzymes that control the redox state of Cys residues and can catalyse thiol-disulfide exchange reactions. The thiol-redox control system comprises the glutathione/glutaredoxin and thioredoxin systems, whose common feature is the utilisation of NADPH as a hydrogen and electron donor [2]. The predominant source of NADPH in *S. cerevisiae* cells growing on a glucose-containing medium is the pentose phosphate pathway (PPP), which involves the conversion of glucose-6-phosphate to ribulose-5-phosphate, concurrently reducing two molecules of NADP^+^ to NADPH. These reactions are catalysed by glucose-6-phosphate dehydrogenase (G6PDH, Zwf1p, product of *ZWF1* gene) and 6-phosphogluconate dehydrogenase (6-PGD, Gnd1p and Gnd2p, product of *GND1* and *GND2* genes) [16,17,18]. An essential source of NADPH is also the NADP^+^-dependent oxidation of acetaldehyde to acetate via aldehyde dehydrogenase 6 (Ald6p, encoded by the *ALD6* gene) [19].

This study aimed to examine how *S. cerevisiae* cells defective in the PPP-dependent NADPH generation (due to deletion of *ZWF1*, *GND1*, or *GND2* genes), respond to redox homeostasis disruption caused by allyl alcohol (AA), a metabolic precursor of acrolein. Allyl alcohol is oxidized intracellularly to acrolein by alcohol dehydrogenase (ADH) [12,20,21,22], and the exposure of the yeast to allyl alcohol leads to a considerable increase in acrolein content inside the cells [12,23]. Replacement of direct usage of acrolein by allyl alcohol introduction allows for using lower concentrations of toxin and elimination of extracellular effects of acrolein resulting from its high reactivity [11,23]. Moreover, the use of *S. cerevisiae* yeast as an experimental model has an advantage resulting from lack of endogenous source of acrolein coming from lipid peroxidation, what was proposed by our team [23] and has been used in subsequent studies [9,10,11,12,14]. Such experimental approach allows to investigate the importance of different NADPH production strategies that may occur in yeast cells under conditions of disrupted redox homeostasis caused by allyl alcohol/acrolein action. The investigation included an assessment of growth capacity, redox parameters, mitochondrial activity, and redox-related gene expression, including genes involved in glutathione and NADPH metabolism, in yeast cells exposed to allyl alcohol.

## 2. Results

### 2.1. Yeast Mutants Defective in PPP-Dependent NADPH Generation Are Sensitive to Acrolein

**Figure 1:**
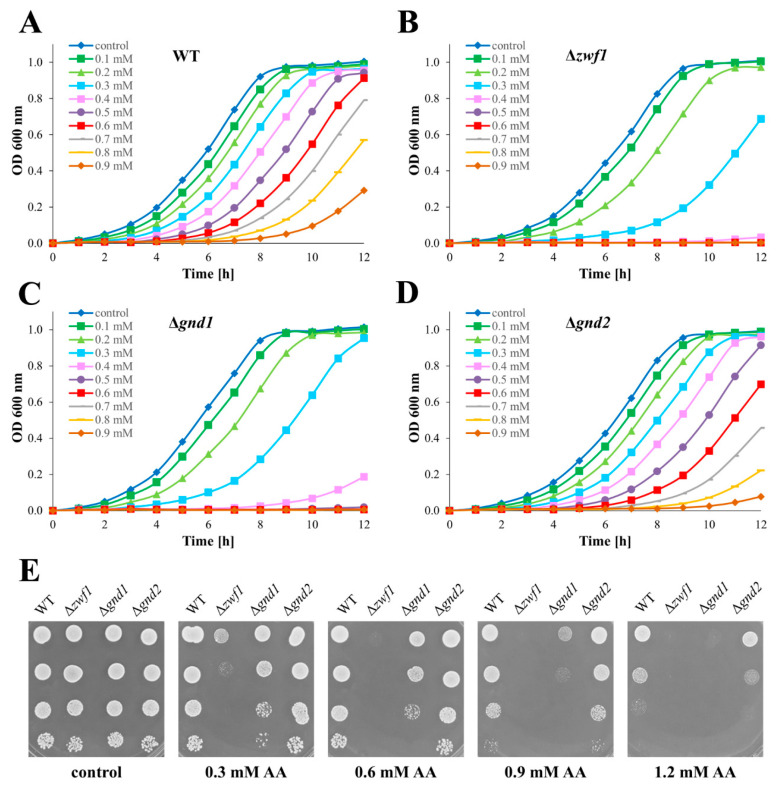
Growth of the WT and pentose phosphate pathway mutant strains cultured with increasing concentrations of allyl alcohol (AA). Growth curves of WT (**A**), Δ*zwf1* (**B**), Δ*gnd1* (**C**), and Δ*gnd2* (**D**) strains cultured in liquid YPD medium supplemented with increasing concentrations of AA. Growth was monitored spectrophotometrically at OD 600 nm during 12 h. The growth curves present the mean from three independent experiments in each case. Spot assay (**E**) showing growth on solid YPD medium containing increasing concentrations of AA. The drops contain 50,000, 5000, 500, and 50 cells, respectively. The growth on solid YPD medium was examined after 48 h, and the pictures show representative views.

Disruption to cellular redox homeostasis caused by a lack of enzymes responsible for the PPP-dependent production of NADPH has several consequences that are crucial for the cell’s efficient functioning. This relates to the strategies that cells use to maintain an appropriate NADPH pool and intracellular redox homeostasis [3,24]. Furthermore, the observed redox disturbances and compensatory mechanisms may vary depending on the metabolic state of the cells, e.g., the growth phase [3]. Thus, it is reasonable to question the sensitivity and ability to maintain the redox homeostasis by strains with disruption in PPP-dependent NADPH generation exposed to allyl alcohol (a metabolic precursor of acrolein). It has been previously shown that the absence of PPP enzymes involved in NADPH generation (mainly Zwf1p and Gnd1p) results in a reduction in cell growth, but only during the exponential phase of culture [3,24]. The growth of the yeast strains defective in NADPH production in the oxidative branch of the PPP, cultured in the presence of increasing concentrations of allyl alcohol, confirmed previous dependencies, but at the same time, allowed us to determine the importance of individual genes, which cannot be observed under optimal conditions (Figure 1). The kinetic growth of the yeast cells cultivated in the liquid medium (Figure 1A–D) revealed a difference in tolerance to acrolein formed intracellularly from allyl alcohol among the analysed strains. The WT cells exhibited growth under almost all tested allyl alcohol concentrations; significant growth inhibition and failure to reach the plateau phase were observed at a concentration of 0.7 mM AA (Figure 1A). The Δ*zwf1* mutant showed the most pronounced growth defect, with significant growth inhibition and failure to reach the plateau phase observed already at a concentration of 0.3 mM AA (Figure 1B). The Δ*gnd1* mutant displayed high sensitivity to allyl alcohol, but less severe than the Δ*zwf1* strain, suggesting partial compensation by Gnd2p (Figure 1C). In turn, the Δ*gnd2* mutant exhibited a growth profile only slightly weaker than the WT, confirming that Gnd1p is the major isoform of yeast 6-phosphogluconate dehydrogenase (Figure 1D). The results of the spot assay (Figure 1E), analysing the growth of cells on solid medium containing increasing concentrations of allyl alcohol, corroborate the findings from liquid culture. The WT and Δ*gnd2* strains maintained the capacity of colony formation across almost all tested allyl alcohol concentrations. The Δ*gnd1* mutant showed reduced colony size and number at higher allyl alcohol concentrations (more than 0.6 mM). In contrast, the Δ*zwf1* strain was unable to grow already at 0.3 mM allyl alcohol (Figure 1E). These results demonstrate that strains with impaired NADPH production in the pentose phosphate pathway are hypersensitive to acrolein formed intracellularly from allyl alcohol, with the *ZWF1* gene deletion causing the most severe phenotype upon long-term exposure to allyl alcohol.

### 2.2. Deletion of the GND1 Gene Has More Severe Growth Repercussions than Deletion of the ZWF1 Gene in Conditions of Short-Term Allyl Alcohol Exposure

To identify the cellular changes linked to growth limitations in cells with impaired NADPH production in the PP pathway, exposed to allyl alcohol, and determine whether these are caused by cell death or inhibited proliferation, appropriate analyses were carried out. For this purpose, yeast cells were incubated with allyl alcohol. The incubation time of 1 h and the 0.6 mM allyl alcohol concentration were chosen based on screening tests and our previous research on acrolein toxicity in yeast [11,14].

**Figure 2:**
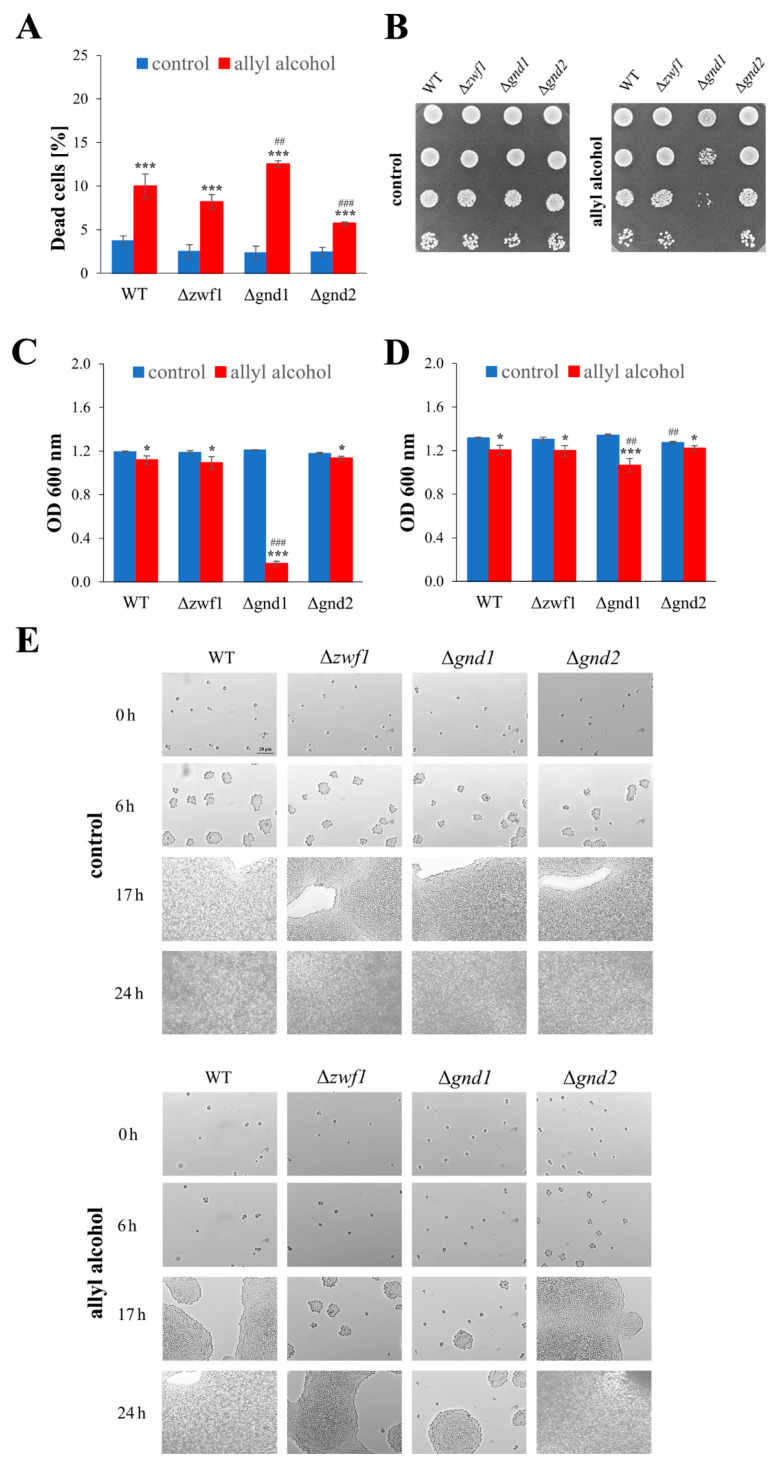
The growth, viability, and vitality of the WT and pentose phosphate pathway mutant strains after 1 h treatment with 0.6 mM allyl alcohol (AA). The viability of the cells (**A**) was estimated with propidium iodide fluorescence staining after 1 h of treatment with 0.6 mM AA. The growth of yeast cells (WT and mutants) on a solid YPD medium (**B**) after 1 h of treatment with 0.6 mM AA. The growth was examined after 48 h. The drops contain 50,000, 5000, 500, and 50 cells, respectively. The growth (OD 600 nm measurements) of yeast cells, previously treated with 0.6 mM AA, after 17 h (**C**) and 24 h (**D**) of the culture on a liquid YPD medium. The viability and budding ability of yeast cells (**E**), previously treated with 0.6 mM AA, examined by microscopic analysis of cells growing on YPD medium plates with phloxine B. The pictures show representative views after the indicated time. The results are presented as the mean ± SD from at three independent experiments in each case. Used designations: * *p* < 0.05, *** *p* < 0.001 comparing cells treated with allyl alcohol vs. untreated control cells; ^##^ *p* < 0.01, ^###^ *p* < 0.001 comparing mutants vs. WT.

The results of the cell viability assay using propidium iodide (PI) dye showed increased death rate of the cells exposed to allyl alcohol in all analysed strains (Figure 2A). Although the percentage of dead cells did not exceed 15%, the Δ*gnd1* strain unexpectedly exhibited the highest mortality rate (Figure 2A). Additionally, the analysis of cell growth after 1 h of incubation in the presence of allyl alcohol, followed by growth on solid (Figure 2B) or liquid medium (Figure 2C,D) without this compound, revealed the most significant growth disturbances in the case of the Δ*gnd1* strain (Figure 2B–D). The growth of all analysed strains was slightly decreased after exposure to allyl alcohol compared to untreated control cells (Figure 2B–D). However, the most significant problems in achieving high population density were noted for the Δ*gnd1* strain (Figure 2C,D), not the Δ*zwf1* strain, as was observed in the case of cells cultured in the constant presence of allyl alcohol (Figure 1). The observed disturbances in the growth of the Δ*gnd1* strain cells after a 1 h exposure to allyl alcohol were the result of blocking their budding ability, as confirmed by microscopic observation of cells treated previously with allyl alcohol and subsequently re-inoculated into a rich solid medium (Figure 2E). The slower growth and the need for an extended time to return to budding after allyl alcohol exposure have also been observed for the Δ*zwf1* strain (Figure 2E). These results demonstrate that deletion of the *ZWF1* or *GND1* genes causes different phenotypes upon long-term and short-term exposure to allyl alcohol.

### 2.3. Short-Term Allyl Alcohol Exposure Disrupts Mitochondria Activity and Modifies Cellular Metabolism, Especially in the Case of Deletion of the GND1 Gene

**Figure 3:**
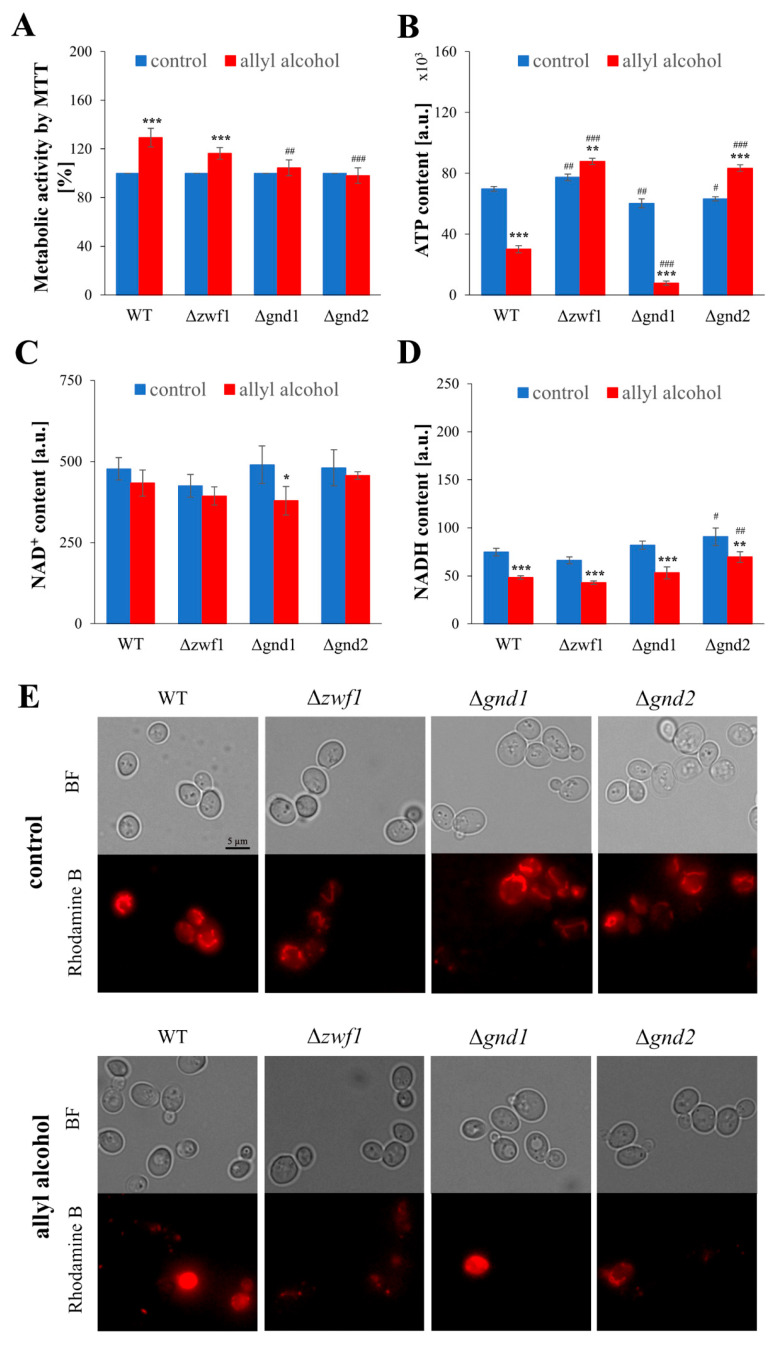
Metabolically associated parameters and mitochondrial activity in the WT and pentose phosphate pathway mutant strains after 1 h treatment with 0.6 mM allyl alcohol (AA). Metabolic activity of the cells (**A**) estimated by MTT assay after 1 h of treatment with 0.6 mM AA. Intracellular ATP content (**B**) determined with BacTiter-Glo Microbial Cell Viability Assay after 1 h of treatment with 0.6 mM AA. The content of pyridine nucleotide cofactors NAD^+^ (**C**) and NADH (**D**) determined with NAD^+^/NADH-Glo Assay in cells previously treated with 0.6 mM AA for 1 h. Mitochondrial activity and morphology of the mitochondrial network (**E**) determined using rhodamine B hexyl ester. Mitochondrial network was visualised using a fluorescence microscope Olympus BX-51 equipped with the DP-72 digital camera and cellSens Dimension v1.0 software at λ_ex_ = 555 nm and λ_em_ = 579 nm. The microscopic images present typical results from the duplicate experiment. Magnification 100×. The results are presented as the mean ± SD from at three independent experiments. Used designations: * *p* < 0.05, ** *p* < 0.01, *** *p* < 0.001 comparing cells treated with allyl alcohol vs. untreated control cells; ^#^ *p* < 0.05, ^##^ *p* < 0.01, ^###^ *p* < 0.001 comparing mutants vs. WT.

Blocking the budding ability of the cells after exposure to allyl alcohol appears to be connected with changes in cell metabolism. This is highly plausible given that proliferation requires appropriate expenditure of energy and building blocks. It can be observed that several metabolic-associated parameters are significantly changed after allyl alcohol exposure (Figure 3). The metabolic activity, as determined by the MTT assay, increased in both the WT and the Δ*zwf1* strains (Figure 3A), suggesting a mitochondria-associated response to acrolein formed intracellularly from allyl alcohol. This is likely because the MTT assay is based on the reduction activity of mitochondrial dehydrogenases. The determined ATP level showed the difference in energy generation abilities in the analysed strains after exposure to allyl alcohol (Figure 3B). The ATP content decreased in cells of the WT and the Δ*gnd1* strains after their exposure to allyl alcohol. However, an enormous reduction in ATP content, as much as 90% was observed in the case of the Δ*gnd1* strain (Figure 3B). The decrease in ATP content appears to correlate with the disruption of the mitochondrial network after allyl alcohol, with the most significant disturbances observed in both the WT and the Δ*gnd1* strains (Figure 3E).

However, at the same time, the ATP content in the case of Δ*zwf1* and Δ*gnd2* strains slightly increased after exposure to allyl alcohol (Figure 3B), despite the observable disorders in mitochondrial activity (Figure 3E). There are also observable changes in the level of NAD(H) cofactors produced in central carbon metabolism pathways and required in the electron transport chain (ETC) as a source of reducing power. The level of NADH decreased in all analysed strains after their exposure to allyl alcohol (Figure 3D), whereas the NAD^+^ significantly declined only in the case of the Δ*gnd1* strain (Figure 3C). As NAD^+^ is a cofactor used by alcohol dehydrogenases (mainly Adh1p) for the conversion of allyl alcohol to acrolein [12], the expression of the *ADH1* gene in cells exposed to 1 h incubation of allyl alcohol was determined. It can be observed that the expression of the *ADH1* gene decreased in all analysed strains after allyl alcohol exposure, except for the Δ*gnd1* strain, where its expression remarkably increased (Figure S1). The most minor changes in metabolic-associated parameters were observed in the case of the Δ*gnd2* strain (Figure 3). These results demonstrate that short-term exposure to allyl alcohol disrupts mitochondrial activity and modifies cellular metabolism to varying extents, depending on the type of disruption in PPP-dependent NADPH generation. The *GND1* gene deletion causes the most severe disruption. The adverse effects of the *GND1* gene deletion in the case of short-term allyl alcohol exposure appear to be partly linked to the possible faster formation of acrolein due to oxidative reactions catalysed by alcohol dehydrogenases, including Adh1p.

### 2.4. Acrolein-Induced Oxidative Stress Is Caused by Glutathione Depletion and Inadequate Redox Compensation in PPP Mutants

**Figure 4:**
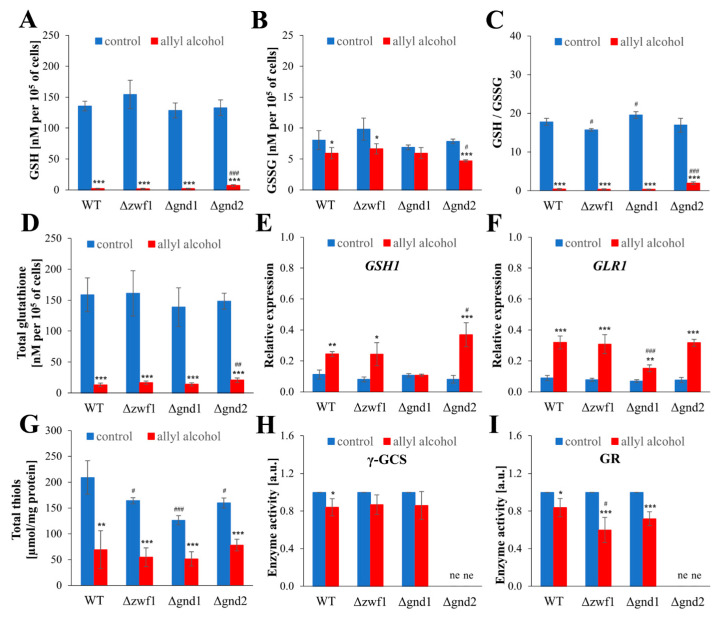
The glutathione content and metabolism in the WT and pentose phosphate pathway mutant strains after 1 h treatment with 0.6 mM allyl alcohol (AA). The reduced glutathione (GSH) content (**A**), the oxidised glutathione (GSSG) content (**B**), the GSH/GSSG ratio (**C**), and the total glutathione content (**D**) estimated after 1 h of treatment with 0.6 mM AA with GSH/GSSG-Glo Assay. Glutathione contents were determined using the luciferin/luciferase-based luminescence method (see Section 4 for details). The relative *GSH1* (**E**) and *GLR1* (**F**) gene expressions in yeast cells after 1 h of treatment with 0.6 mM AA. *GSH1* and *GLR1* gene expressions were determined by qPCR assay with TaqMan probes. The relative gene expression was calculated with the −ΔC_T_ method for comparison of the expression of one gene in all tested strains. The thiol group content (**G**) estimated in yeast cells after 1 h of treatment with 0.6 mM AA. Total thiol group content was determined with DTNB (Ellman method) and absorbance measurements at 412 nm. The activity of the γ-GCS (**H**) and GR (**I**) enzymes estimated after 1 h of cells treatment with 0.6 mM AA. The activities of the enzymes were determined in the whole-cell protein extracts. The γ-GCS activity was measured by the reaction coupled with lactic dehydrogenase and pyruvate kinase (PK/LDH enzymes mixture for determination of ADP) by the decrease in NADH absorbance at 340 nm. The GR activity was determined by the rate of NADPH absorbance decrease at 340 nm. The abbreviation ‘ne’ means that the sample was not estimated. The results are presented as the mean ± SD from at least three independent experiments. Used designations: * *p* < 0.05, ** *p* < 0.01, *** *p* < 0.001 comparing cells treated with allyl alcohol vs. untreated control cells; ^#^ *p* < 0.05, ^##^ *p* < 0.01, ^###^ *p* < 0.001 comparing mutants vs. WT.

The reducing intracellular environment is maintained by the glutathione redox buffer and protein thiol maintenance systems, both of which are related to NADPH-generating reactions. Therefore, it was essential to evaluate the glutathione content and metabolism after 1 h of exposure to allyl alcohol in strains deficient in PP pathway enzymes. The level of reduced glutathione (GSH) was comparable in control cells of all strains tested and decreased dramatically, more than ten times (almost to no GSH detectable level) after the exposure of cells to allyl alcohol (Figure 4A), confirming our previous observation [11,14] that acrolein is a strong electrophile depleting the pool of GSH. The loss of reduced glutathione did not involve the concomitant increase in the content of oxidised glutathione (GSSG) (Figure 4B). The level of GSSG was even decreased in cells treated with allyl alcohol in the WT, ∆*zwf1*, and ∆*gnd2* strains, but not in the ∆*gnd1* strain (Figure 4B). This suggests that the formation of acrolein–GSH conjugates mainly causes the loss of glutathione.

**Figure 5:**
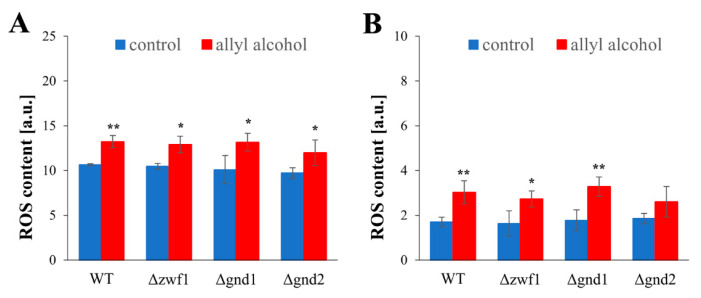
The reactive oxygen species (ROS) content in the WT and pentose phosphate pathway mutant strains after 1 h treatment with 0.6 mM allyl alcohol (AA). The ROS content estimated with DHET (**A**) and H_2_DCF-DA (**B**). The ROS generation was estimated by the rate of fluorescence increase due to dihydroethidium (DHET) or 2′,7′-dichlorodihydrofluorescein (H_2_DCF) oxidation within cells. Measurements were performed for 30 min at 28 °C at λ_ex_ = 518 nm, λ_em_ = 605 nm for DHET and λ_ex_ = 495 nm, λ_em_ = 525 nm for H_2_DCF-DA. The results are presented as the mean ± SD from at least three independent experiments. Used designations: * *p* < 0.05, ** *p* < 0.01 comparing cells treated with allyl alcohol vs. untreated control cells.

These significant changes in GSH and GSSG content, caused by allyl alcohol exposure, also result in a substantial reduction in the redox potential, as expressed by the GSH/GSSG ratio (Figure 4C). The disrupted GSH/GSSG ratio was already noted in the control cells for the ∆*zwf1* strain. Still, after allyl alcohol exposure, the decline in the GSH/GSSG ratio was comparable for all strains tested (Figure 4C). Only a slightly less severe decrease in GSH/GSSG ratio and GSH level was observed in the case of the ∆*gnd2* strain (Figure 4A,C). The observable depletion of GSH (Figure 4A) has led to a significant loss of total glutathione content (Figure 4D). According to those changes, total thiol content (protein sulfhydryl residues and glutathione) was also diminished (Figure 4G) but not so dramatically as the reduced and total glutathione (Figure 4A,D), indicating that glutathione is the main target of the reactivity of acrolein generated from allyl alcohol. This confirms the protective role of glutathione to shield cellular proteins. The total thiols contents were lower in control cells of the strains with disruption in PPP-dependent NADPH generation compared to the WT (Figure 4G). The lowest thiol content in control cells was observed in the ∆*gnd1* strain (Figure 4G). In turn, the thiol content decreased after exposure to allyl alcohol, reaching a similar level in all analysed strains (Figure 4G). The disturbance in cellular glutathione redox buffer caused by the generation of acrolein induced alterations in glutathione metabolism proteins and respective gene expression (Figure 4E,F,H,I). De novo synthesis of GSH is a two-step ATP-dependent reaction of the ligation of amino acids. The first step is catalysed by γ-glutamylcysteine ligase (γ-GCS) encoded by the *GSH1* gene. The basal level of the *GSH1* gene expression was similar in all strains. The induction of the *GSH1* gene expression was apparent after 1 h-exposure to allyl alcohol in the cells of WT, Δ*zwf1*, and Δ*gnd2* strains, but not in the Δ*gnd1* strain, in comparison to control cells, not exposed to allyl alcohol (Figure 4E). The induction of the *GSH1* gene expression was not accompanied by an increase in γ-GCS activity (Figure 4H), which was unaffected or even slightly diminished as in the WT. Another way to counterbalance the loss of glutathione is the reduction in glutathione disulfides by glutathione reductase (GR). The activity of GR was decreased in all tested strains after treatment with allyl alcohol, although the *GLR1* gene was significantly upregulated (Figure 4I,F). The upregulation of the *GLR1* gene expression after allyl alcohol exposure was most strongly marked for the WT, Δ*zwf1*, and Δ*gnd2* strains (about four times higher in relation to control cells), whereas lower upregulation was noted in the case of the Δ*gnd1* strain (Figure 4F). The disturbance of glutathione homeostasis induced by allyl alcohol treatment (Figure 4) was accompanied by an increase in intracellular ROS content (Figure 5). The generation of ROS was measured using two fluorescent probes, dihydroethidine and dichlorofluorescin diacetate. Both measurements revealed a similar effect, namely an equal increase in ROS content after treatment with allyl alcohol in each of the tested strains (Figure 5A,B). No differences in the ROS level were observed in the control cells of the analysed strains, indicating that the ROS level is not affected by the potential for NADPH generation.

### 2.5. NADPH-Mediated Redox Compensation and the Thioredoxin System Are Key Determinants of Stress Response to Acrolein in Yeast Cells

**Figure 6:**
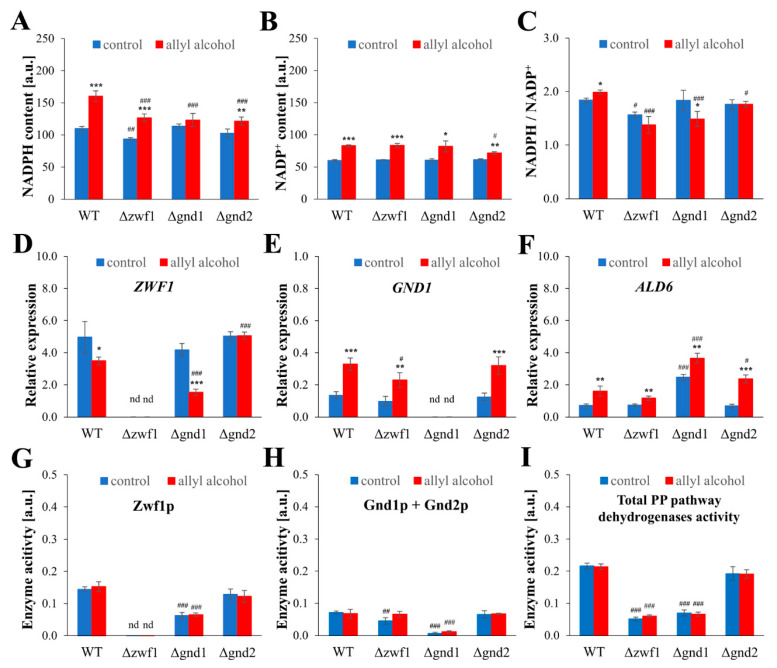
The NADP(H) cofactors content, the gene expression, and the activity of enzymes responsible for fermentation-dependent NADPH generation in the WT and pentose phosphate pathway mutant strains after 1 h treatment with 0.6 mM allyl alcohol (AA). The NADPH content (**A**), the NADP^+^ content (**B**), and the NADPH/NADP^+^ ratio (**C**), estimated after 1 h of treatment with 0.6 mM AA with NADP^+^/NADPH-Glo Assay. NADP(H) contents were determined using the luciferin/luciferase-based luminescence method (see Section 4 for details). The relative *ZWF1* (**D**), *GND1* (**E**), and *ALD6* (**F**) gene expressions in yeast cells after 1 h of treatment with 0.6 mM AA. The *ZWF1*, *GND1*, and *ALD6* gene expressions were determined by qPCR assay with TaqMan probes. The relative gene expression was calculated using the −ΔC_T_ method for comparison of the expression of one gene in all tested strains. The activity of PP pathway dehydrogenases: glucose-6-phosphate dehydrogenase—Zwf1p (**G**), 6-phosphogluconate dehydrogenase—Gnd1p and Gnd2p (**H**), and total PP pathway dehydrogenases (**I**) activity were determined spectrophotometrically in whole-cell extracts by measuring the increase in NADPH absorbance at 340 nm. The abbreviation ‘nd’ means that the determination was performed and the values were not detectable. The results are presented as the mean ± SD from at least three independent experiments. Used designations: * *p* < 0.05, ** *p* < 0.01, *** *p* < 0.001 comparing cells treated with allyl alcohol vs. untreated control cells; ^#^ *p* < 0.05, ^##^ *p* < 0.01, ^###^ *p* < 0.001 comparing mutants vs. WT.

**Figure 7:**
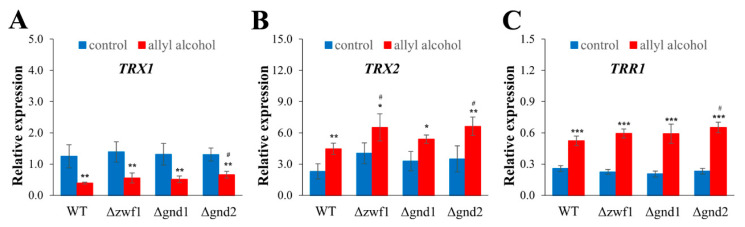
Expression of cytoplasmic thioredoxin system genes in the WT and pentose phosphate pathway mutant strains after 1 h treatment with 0.6 mM allyl alcohol (AA). The relative *TRX1* (**A**), *TRX2* (**B**), and *TRR1* (**C**) gene expressions were determined by qPCR assay with TaqMan probes. The relative gene expression was calculated with the −ΔC_T_ method for comparison of the expression of one gene in all tested strains. The results are presented as the mean ± SD from at least three independent experiments. Used designations: * *p* < 0.05, ** *p* < 0.01, *** *p* < 0.001 comparing cells treated with allyl alcohol vs. untreated control cells; ^#^ *p* < 0.05 comparing mutants vs. WT.

Glutathione and the redox potential of the GSH/GSSG couple play a crucial role in maintaining intracellular redox homeostasis. However, other redox couples, such as NADPH/NADP^+^, may also influence redox homeostasis. NADPH is an essential reducing equivalent donor, necessary for the functioning of the glutathione system (as a cofactor for GR), but also for the thioredoxin reductase (TRR), which is required to maintain reduced thioredoxins (TRXs). Thus, the availability of NADPH is crucial for the efficient reduction in glutathione and maintaining the redox balance in cells, especially under stress conditions. Hence, the intracellular content of NADPH, NADP^+^, and the NADPH/NADP^+^ ratio in cells treated with allyl alcohol was measured. In control conditions, the NADPH/NADP^+^ ratio and NADPH content were decreased only in the cells of the Δ*zwf1* strain (Figure 6A,C). Exposure to allyl alcohol results in a considerable increase in NADPH content: more than 40% in the cells of the WT, more than 30% in the Δ*zwf1* strain, and about 20% in the Δ*gnd2* strain. In contrast, such an increase was not observed in the case of the Δ*gnd1* strain (Figure 6A). Simultaneously, the NADP^+^ content was elevated in all tested strains after treatment with allyl alcohol (Figure 6B), and the ratio of NADPH/NADP^+^ was altered (Figure 6C). The NADPH/NADP^+^ ratio increased in the cells of the WT, decreased slightly in the Δ*zwf1* strain, remained unchanged in the Δ*gnd2* strain, but declined significantly in the Δ*gnd1* strain, in each case compared to control, untreated cells (Figure 6C). As the changes in NADP(H) content were observed and taking into account the differences in compensation mechanisms occurring in cells impaired in NADPH production [3], the expression of *ZWF1*, *GND1*, and *ALD6* genes and the activity of NADPH-producing dehydrogenases after allyl alcohol exposure were determined (Figure 6D–I). Compared to the untreated control cells, the expression of the *ZWF1* gene was downregulated in the WT and Δ*gnd1* strains (Figure 6D). In turn, the expression of the *GND1* gene was significantly upregulated in all analysed strains (Figure 6E), as well as the expression of the *ALD6* gene (Figure 6F). In the case of *ALD6* gene expression, the Δ*gnd1* strain exhibits a considerably higher level of expression, even compared to the control cells (Figure 6F). Further upregulation in the *ALD6* gene expression in the Δ*gnd1* strain after allyl alcohol exposure was noted (Figure 6F). Despite the upregulation of PP pathway gene expression, the level of activity of respective proteins was not changed after allyl alcohol exposure (Figure 6G–I). The critical consumer of cellular NADPH can also be the thioredoxin system, comprising thioredoxins and directly NADPH-dependent thioredoxin reductase. The expression of genes encoding cytosolic thioredoxins (*TRX1* and *TRX2*) and cytosolic thioredoxin reductase (*TRR1*) under the conditions of electrophilic stress induced by acrolein was determined (Figure 7). Treating the cells with allyl alcohol significantly influences the expression of thioredoxin system genes, downregulates *TRX1* gene expression to a similar extent in all tested strains (Figure 7A). In contrast, the *TRX2* gene was considerably upregulated in all tested strains when cells were exposed to allyl alcohol (Figure 7B). Moreover, the level of the *TRX2* transcript is dominant in comparison to other estimated components of the thioredoxin system (Figure 7B). Similarly, the *TRR1* gene expression was also upregulated in all tested strains (Figure 7C). This suggests that the induction of the thioredoxin system after allyl alcohol exposure may be part of the cellular response to allyl alcohol and does not depend on the specific way of NADPH production sourcing.

## 3. Discussion

### 3.1. Disorders of Redox Homeostasis Under Exposure to Acrolein

Acrolein, a reactive aldehyde formed within cells from allyl alcohol, poses a significant threat to the redox balance by rapidly depleting reduced glutathione (GSH), forming of acrolein–GSH conjugates and inducing oxidative stress. The priority of formation of acrolein–GSH conjugates after allyl alcohol exposure can be established by several observations: (i) depletion of GSH and total glutathione level [10,11]; Figure 4 in this paper; (ii) the decrease in total cellular thiols [10]; Figure 4 in this paper; (iii) lack of GSH oxidation or even lower level of GSSG content [10]; Figure 4 in this paper; (iv) increased activity [10] and expression of glutathione-S-transferases, i.e., the *GTT2* gene [9]. The glutathione depletion, which occurs within a few minutes of exposure to allyl alcohol, suggests its protective role in shielding cellular thiol groups from oxidation (Figure 4G). However, depletion of GSH and the tremendous disruption of glutathione redox potential (Figure 4C) result in a redox imbalance, leading to oxidative stress [11,14]; this paper. Hence, acrolein generated from allyl alcohol activates Yap1 transcription factor and cellular stress response [11,25]. Moreover, after a longer time of allyl alcohol treatment, but not directly after addition [11,14], the increased ROS generation can be observed [14]; Figure 5 in this paper. This suggests that the ROS generation is a secondary effect of acrolein toxicity. Nevertheless, it plays a significant role in the case where strains have disruption directly in the antioxidant defence system, as demonstrated by the Δ*sod1*, Δ*gsh1*, and Δ*trx1*Δ*trx2* mutants that are hypersensitive to allyl alcohol [11,12,14]. This type of protective role of glutathione against acrolein toxicity may, however, be insufficient, as indicated by our previous results, showing enormous protein carbonylation induced by acrolein in the WT and Δ*sod1* yeast cells [14].

### 3.2. Response Mechanisms and Possible Adaptations Under Acrolein Exposure Conditions

To constrain the negative consequences of short-term allyl alcohol exposure and intracellularly generated acrolein, cells activate a cellular stress response. The results of this work and previous literature data indicate that this response may be multidimensional (Table S1), and occurs through: (i) attempts to increase glutathione synthesis; (ii) changes in the thioredoxin system; (iii) cell cycle and biosynthetic processes arrest; and (iv) shown for the first time (to author’s knowledge), stimulation of NADPH synthesis. It is worth noting that particular responses are interrelated, can complement each other, and their action among others is dependent on transcription factor Yap1 activity. Yap1p is a b-ZIP protein composed of two cysteine-rich domains (N-CRD and C-CRD), and there are two ways of Yap1 activation, i.e., Gpx3-mediated oxidation of cysteines and forming intramolecular disulfide bonds between CRDs or direct oxidation of C-CRD cysteines by electrophilic compounds [26,27]. It was previously shown that acrolein activates Yap1p in the Δ*gpx3* strain, revealing that acrolein-dependent Yap1 activation occurs by direct oxidation of Yap1 cysteines [11]. Among several genes whose expression is induced in a Yap1p-dependent fashion, it is essential to mention the genes of the thioredoxin and glutathione systems, including the *GSH1*, *GLR1*, *TRX2*, and *TRR1* genes [28,29]. The upregulated expression of glutathione metabolism genes—the *GSH1* and *GLR1* after allyl alcohol exposure, found in this work (Figure 4) and previously [9], indicates that cells try to rebuild the worn-out glutathione buffer system. Although the upregulation of *GSH1* and *GLR1* gene expression did not correspond with an instant increase in activity of respective proteins (Figure 4E–I), still several reports indicate the reducing acrolein toxicity by increasing the level of glutathione and thiol compounds [9,10,11,13,15]. It was found that exogenously added thiol antioxidants, such as glutathione, *N*-acetylcysteine, cysteine, and dithiothreitol, reduce the sensitivity of yeast mutant strains to allyl alcohol and can protect their proteins against oxidation [11,13]. The transcriptome analysis in cells exposed to allyl alcohol revealed that, in addition to the upregulated expression of the *GSH1* and *GLR1* genes, attempts to increase glutathione synthesis can also be linked to a higher cysteine supply, as genes involved in sulphate assimilation and biosynthesis of homocysteine were significantly upregulated [9]. Moreover, strains with higher resistance to acrolein obtained through adaptive laboratory evolution (ALE) exhibit improved glutathione production [15].

Besides the glutathione system, the thioredoxin system is another crucial player in maintaining redox homeostasis. The role of the thioredoxins is to reduce key Cys residues in several intracellular proteins, including peroxiredoxins, ribonucleotide reductase, protein disulfide isomerase, and methionine sulfoxide reductase [30,31]. Yeast cytosolic thioredoxins (Trx1p and Trx2p) are reduced by NADPH-dependent thioredoxin reductase (Trr1p). Our results show that short-term exposure to allyl alcohol downregulates the expression of the *TRX1* gene, but significantly upregulates the expression of the *TRX2* and *TRR1* genes (Figure 7). The explanation for this observation is not simple, but it may be related to the slightly different roles of Trx1p and Trx2p in the yeast cell [32,33]. Trx1p and Trx2p are not in redox equilibrium with each other [34]. Trx2p preferentially interacts with the cytoplasmic peroxiredoxins Ahp1p and Tsa1p, which are involved in oxidative stress reduction, whereas Trx1p preferentially interacts with PAPS reductase (Met16p), which is involved in sulphur metabolism [33]. The *TRX2* gene expression is more readily induced under oxidative stress conditions, while the *TRX1* gene expression remains relatively stable. Likewise, the Δ*trx2* mutant is hypersensitive to oxidants, while the Δ*trx1* mutant is not [32]. Some of the results concerning mammalian thioredoxin proteins also indicate that they can be oxidised by micromolar concentrations of acrolein [34]. In human epithelial cells, Trx1p was found to be more susceptible to oxidation by acrolein [35]. In conjunction with our results, this indicates that in the yeast cells, Trx2p plays an essential role in acrolein-induced stress response and thus can be critical for oxidative stress and redox perturbation defence. Upregulation of cytosolic thioredoxin system (*TRX2* and *TRR1* genes) induced by acrolein may arise from (i) induced expression of these genes by Yap1 transcription factor; (ii) depletion of total cellular thiols and increased protein carbonylation; (iii) potential inhibition of thioredoxin system proteins by acrolein; (iv) physiological role of Trx2p in Yap1p reduction (Trx2p is a reductant of oxidised form of Yap1p necessary to break the stress response). It is worth noting that higher expression of the *TRR1* gene can also be connected with lower GSSG levels observed after exposure to allyl alcohol (Figure 4B), as the possibility of GSSG reduction by Trr1p is a well-known phenomenon [36].

Both the glutathione reduction system and the activity of thioredoxins are closely related to the redox-active cofactors system, specifically in terms of the level of reduced NADPH and the NADPH/NADP^+^ ratio. Currently, more and more data carried out both on yeast cells [3,10,37]; this paper and on cell lines [38] demonstrate the existence of connection, balancing, and rapid equilibration of the glutathione and NADP(H) redox couples. In the present work, we show for the first time (to the author’s knowledge) that stimulation of NADPH synthesis may also be an essential cell response to maintain redox homeostasis and cope with acrolein toxicity. It has been observed that after exposure to allyl alcohol, yeast cells increased the level of NADPH (Figure 6A), induced the expression of the *GND1* and *ALD6* genes (Figure 6E,F), and maintained the activity of the PP pathway NADP^+^ dehydrogenases (Figure 6G–I). The cellular significance of these changes resulting in increased NADPH levels may be related to (i) increased NADPH demands to reduce glutathione disulfide (Figure 4); (ii) increased NADPH demands for the thioredoxin system (Figure 7); (iii) involvement of NADPH/NADP^+^ couples to counterbalance the drop in GSH/GSSG ratio (Figure 4 and Figure 6); (iv) involvement of NADPH in acrolein detoxification reactions and its conversion catalysed by conserved NADPH-dependent oxidoreductases (mainly Oye2p) [39]. Although the leading role in the conversion of acrolein to propionaldehyde is found to be served by Oye2p (a mutant lacking the *OYE2* gene, but not the *OYE3* gene, was sensitive to acrolein), overexpression of both isoenzymes increased acrolein tolerance [9,39]. The increased level of NADP^+^ observed after exposure to allyl alcohol indicates the successive utilisation of the formed NADPH. Nevertheless, the increased NADPH content observed in these conditions can also be partly attributed to the lower usage of NADPH for cellular biosynthetic needs. After allyl alcohol exposure, disturbances in cell growth can be observed, resulting not from a highly increased cell mortality, but from the blockage of their budding ability (Figure 2). This corresponds to the results of the transcriptome analysis in cells exposed to allyl alcohol, which showed that expression of ribosomal protein genes, and genes involved in ribosome biogenesis, cell cycle, biosynthesis of vitamins, lipids, and fatty acid metabolism were significantly downregulated [9].

### 3.3. The Strategy of Supplying NADPH in Strains Defective in the PP Pathway Does Not Work When Cells Are Exposed to Allyl Alcohol

Considering the cellular defence response to acrolein, including the aspect related to increased NADPH production, an important goal of this study was to investigate how strains defective in PPP-dependent NADPH generation cope under these conditions. It can be observed that yeast strains defective in PPP-dependent NADPH generation (mainly the Δ*zwf1* and the Δ*gnd1* strains) are sensitive to acrolein. However, their sensitivity varies depending on whether they are exposed to allyl alcohol in the long term or short term (Figure 1 and Figure 2). Our previous studies have shown that the most severe consequences for yeast cells result from the deletion of the *ZWF1* gene [3,24]. Indeed, the highest sensitivity of the Δ*zwf1* strain to acrolein was also observed in the present work, but only during long-term exposure to allyl alcohol (growth in the presence of allyl alcohol) (Figure 1). Whereas during short-term exposure to allyl alcohol, when the cell can trigger a stress response, the Δ*gnd1* strain showed the greatest sensitivity (Figure 2). The obtained results for the Δ*gnd1* strain showed that deletion of the *GND1* gene causes the ways of cellular responses to acrolein (described above) to be ineffective (Figure 3, Figure 4, Figure 5 and Figure 6), and, in addition, the strategy of supplying NADPH undertaken by this strain may even increase the toxicity of allyl alcohol (Figure S1). The observed increased sensitivity of the Δ*gnd1* mutant in the case of short-time exposure to allyl alcohol may have several causes: (i) weaker antioxidant equipment of the Δ*gnd*1 mutant cells; (ii) ineffective response of the Δ*gnd1* strain to stress induced by allyl alcohol, especially in the context of increased glutathione synthesis and stimulation of NADPH synthesis; (iii) consequences of increasing expression of the *ALD6* gene as a strategy of the Δ*gnd1* strain to provide proper NADPH level; (iv) upregulation of the *ADH1* gene expression in the Δ*gnd1* mutant cells exposed to allyl alcohol. The cells of the Δ*gnd1* strain not only show altered NADPH/NADP^+^ and GSH/GSSG ratios, but also, which could be significant in the context of acrolein toxicity, the *GND1* gene deletion leads to reduced levels of GSH, GSSG, total cellular thiols, NADPH, and pyridoxine even in cells grown under normal conditions [3,24]; this paper. The *GND1* gene deletion results in a reduced ability of the cells to restore glutathione depletion induced by acrolein. The expression of the *GSH1* and *GLR1* genes did not change or was upregulated to a lesser extent, respectively, compared to the other tested strains (Figure 4E,F). The activity of the γ-GCS and GR enzymes also decreased slightly (Figure 4H,I); thus, neither increased de novo GSH synthesis nor sufficient GSSG reduction to GSH occurs under stress conditions in this mutant. The lower capacity of glutathione synthesis in the Δ*gnd1* strain is also indicated by the most severe disruption of mitochondrial activity, extremely low ATP content (Figure 3), and decreased level of pyridoxine [24]. De novo synthesis of GSH is catalysed in a two-step ATP-mediated reaction. It depends on the cysteine content, which is generated by the conversion of homocysteine catalysed by two pyridoxine-dependent reactions [2,24,40]. Furthermore, the NADPH pool did not increase as in the other tested strains, and additionally, the NADPH/NADP^+^ ratio significantly decreased in the Δ*gnd1* strain exposed to allyl alcohol (Figure 6A–C). The lack of an increased NADPH content under stress conditions may have several consequences for the cells, including a lower level of GSSG reduction catalysed either by GR or Trr1p, insufficient protection of proteins by the NADPH-dependent thioredoxin system, or lower conversion of acrolein catalysed by NADPH-dependent Oye2p [39]. At the same time, a severe problem for the Δ*gnd1* mutant cells under conditions of exposure to allyl alcohol is the strategy that this strain uses to ensure a proper NADPH level, i.e., NADP^+^-dependent oxidation of acetaldehyde to acetate via Ald6p [3]; Figure 6 in this paper. The use of this pathway for obtaining NADPH is associated with the production of a high level of acetic acid, a by-product that must be removed from the cells by ATP-dependent systems. Therefore, in the Δ*gnd1* mutant cells exposed to allyl alcohol, the upregulation of *ALD6* gene expression, necessary for NADPH formation, can be concomitantly linked to a drastic decrease in ATP content (Figure 3B and Figure 6F). A relevant difference in the *ADH1* gene expression in cells exposed to allyl alcohol was also noted (Figure S1). The expression of the *ADH1* gene decreased in all analysed strains after short-term allyl alcohol exposure, while the Δ*gnd1* mutant cells significantly upregulated the expression of the *ADH1* gene (Figure S1). Higher expression of the *ADH1* gene may be responsible for increased formation of Adh1p, and consequently for higher levels of acrolein generated in cells in a shorter time. Therefore, the toxic effects of acrolein (after short-term exposure to allyl alcohol), such as higher mortality, arrest of cell cycle and blocking budding ability of the cells, growth inhibition, mitochondrial activity reduction, or decreased ATP level, were the most significant in the Δ*gnd1* strain in comparison to the other tested strains (Figure 2 and Figure 3, Table S1). Especially since other tested strains, by downregulation of the *ADH1* gene expression, can probably reduce the rate of acrolein formation from allyl alcohol. The importance of this possibility is indicated by the work of Plapp et al. [20], which presented that yeast with lower sensitivity to allyl alcohol had mutations in the *ADH1* gene that resulted in decreased ADH activity. Thus, these yeasts could grow in the presence of allyl alcohol due to slower oxidation of allyl alcohol, which was also connected with an alteration in the NADH/NAD^+^ ratio [20,41].

## 4. Materials and Methods

### 4.1. Yeast Strains, Growth and Incubation Conditions

In this study, the wild-type strain (WT) BY4742 MATα *his3 leu2 lys2 ura3*, along with three mutant strains isogenic to BY4742: Δ*zwf1* YNL241c::kanMX4, Δ*gnd1* YHR183w::kanMX4, and Δ*gnd2* YGR256w::kanMX4, were used (EUROSCARF, Scientific Research and Development GmbH, Oberursel, Germany). The yeast cultures were cultivated in standard liquid YPD medium, comprising 1% Yeast Extract, 1% Yeast Bacto-Peptone, and 2% glucose, on a rotary shaker set at 150 rpm, or on solid YPD medium containing 2% agar, maintained at a temperature of 28 °C.

For the allyl alcohol treatment (short-time exposure), cells from an exponential phase culture (~17 h; density of approximately 7 × 10^7^ cells/mL) were centrifuged, washed twice, and resuspended to a final density of 1 × 10^8^ cells/mL in a 100 mM phosphate buffer at pH 7.0, containing 1 mM EDTA and 0.1% glucose. The cells were then incubated with 0.6 mM allyl alcohol for 1 h at 28 °C with shaking. Allyl alcohol (AA; CAS number 107-18-6, ≥99%, Sigma-Aldrich, Poznan, Poland) was dissolved in sterile water to prepare a 100 mM stock solution. Following incubation, the cells were centrifuged, washed twice, and prepared for subsequent analysis.

### 4.2. Cell Growth Assays

The growth of cells in a liquid YPD medium with varying concentrations of allyl alcohol (0.1–0.9 mM) was assessed by measuring OD at 600 nm using the Anthos 2010 type 17550 microplate reader (Biochrom Ltd., Cambridge, UK) over 12 h (with measurements taken every 1 h) at 28 °C. The initial density of the yeast suspension was 5 × 10^6^ cells/mL. This analysis was also performed for the cells incubated with 0.6 mM allyl alcohol for 1 h. In this case, the cells were cultivated in a liquid YPD medium (without the addition of allyl alcohol), and OD measurements were recorded at appointed times of cultivation.

For the spotting test, the cells were diluted to create suspensions containing 10^7^, 10^6^, 10^5^, and 10^4^ cells/mL. Aliquots of 5 μL from each dilution were inoculated onto a solid YPD medium containing 0.3, 0.6, 0.9, and 1.2 mM concentrations of allyl alcohol. Colony growth was inspected after 48 h. This analysis was also performed for the cells incubated with 0.6 mM allyl alcohol for 1 h. In this case, the cells were grown on a solid YPD medium without the addition of allyl alcohol.

Cell growth was also monitored by microscopic observation of the cells treated with 0.6 mM allyl alcohol and spread on a solid YPD medium containing phloxine B (10 µg/mL; Sigma-Aldrich, Poznan, Poland). Growth of the cells was observed directly after inoculation and after appointed times (6, 17, and 24 h) using a Nikon Eclipse E200 microscope (Nikon, Tokyo, Japan) fitted with a 20× lens and an Olympus DP10 digital camera (Olympus, Tokyo, Japan).

### 4.3. Cell Viability and Vitality Assays

After 1 h of incubation with 0.6 mM allyl alcohol, the cells were stained with propidium iodide (PI, 5 µg/mL; Molecular Probes, Eugene, OR, USA) [42] for 20 min, and observed using an Olympus BX-51 fluorescence microscope (Olympus, Hamburg, Germany). The number of dead cells (PI-positive) was expressed as a percentage of at least 200 cells in each biological replicate.

The metabolic activity of the cells after a 1 h treatment with 0.6 mM allyl alcohol was determined using Thiazolyl Blue Tetrazolium Bromide (MTT; Sigma-Aldrich, Poznan, Poland) for 30 min. The MTT assay is based on the conversion of MTT into formazan crystals, among other by mitochondrial dehydrogenases in metabolically active cells. The formazan was then solubilised in dimethyl sulfoxide and measured using an Infinite 200 microplate reader (Tecan Group Ltd., Männedorf, Switzerland) at λ = 560 nm. Metabolic activity was expressed as a percentage of the control.

### 4.4. Assessment of Cellular ATP Content

The level of ATP in the cells treated with 0.6 mM allyl alcohol for 1 h was determined using the BacTiter-Glo Microbial Cell Viability Assay (Promega, Madison, WI, USA), according to the manufacturer’s protocol with own modifications [43]. The cells were suspended in a 100 mM phosphate buffer, pH 7.0, containing 0.1% glucose and 1 mM EDTA. A cell suspension sample with a density of 10^6^ cells/mL was used for determination purposes. Once the luminescence had achieved a stable value, the luminescent signal, proportional to the amount of ATP, was recorded using an Infinite 200 microplate reader (Tecan Group Ltd., Männedorf, Switzerland).

### 4.5. Estimation of Mitochondrial Activity and Morphology of the Mitochondrial Network

Mitochondrial activity and the mitochondrial network morphology were determined using rhodamine B hexyl ester (Molecular Probes, Eugene, OR, USA), the fluorescence of which depends on the mitochondrial membrane potential. Cells treated with 0.6 mM allyl alcohol for 1 h were washed twice with sterile water and suspended in a 10 mM HEPES buffer, pH 7.4, containing 5% glucose. The cells were then incubated with 100 nM rhodamine B in the dark at 28 °C for 20 min. After incubation, the mitochondrial network was visualised using fluorescence microscopy at λ_ex_ = 555 nm and λ_em_ = 579 nm. Microscopic images presenting typical results from one of two experiments were captured using a BX-51 microscope equipped with a DP-72 digital camera (Olympus, Tokyo, Japan) and cellSens Dimension v1.0 software.

### 4.6. Determination of Glutathione and Thiol Content

Total glutathione (the sum of GSH and GSSG) and GSSG levels were determined in the yeast cells using the GSH/GSSG-Glo Assay (Promega, Madison, WI, USA), following the manufacturer’s protocol and own modifications [24]. After 1 h incubation with 0.6 mM allyl alcohol, the cell density (number of cells per mL) was determined using a Malassez chamber. The cells were centrifuged, washed twice with sterile water, and suspended in a PBS buffer at a 5 × 10^5^ cells/mL density. The luminescent signal was recorded after 15 min using an Infinite 200 microplate reader (Tecan Group Ltd., Männedorf, Switzerland). Total glutathione and GSSG concentrations were determined using standard curves, whereas GSH levels were calculated by subtracting the GSSG from the total glutathione concentration (as 1 mole of GSSG is generated from 2 moles of GSH, GSSG values were multiplied by 2).

The total thiol content of the cell extract was estimated using 5,5′-dithiobis-(2-nitrobenzoic acid) (DTNB; 10 mg/mL stock solution in 0.5% NaHCO_3_) in a 100 mM Tris-HCl buffer, pH 8.2, containing 1 mM EDTA [44]. Absorbance was measured after 20 min of incubation, at room temperature, using an Infinite 200 microplate reader (Tecan Group Ltd., Männedorf, Switzerland) at λ = 412 nm. An extinction coefficient of 13.6 mM^−1^ cm^−1^ was used to calculate the thiol concentration in the protein extract.

### 4.7. Determination of ROS Content

The generation of reactive oxygen species was assessed using dihydroethidium (DHET; final concentration of 10.7 μM; stock solution in dimethyl sulfoxide; Molecular Probes, Eugene, OR, USA) and 2′,7′-dichlorodihydrofluorescein diacetate (H_2_DCF-DA; final concentration of 15.8 μM; stock solution in ethanol; Sigma-Aldrich, Poznan, Poland). DHET is oxidised by superoxide anion to form the fluorescent compound 2-hydroxyethidium. However, it can also undergo non-specific oxidation to form ethidium in the presence of other oxidants, e.g., the hydroxyl radical, peroxynitrite, and perferryl iron [45,46]. In contrast, after entering the cell, H_2_DCF-DA is hydrolysed, releasing 2′,7′-dichlorodihydrofluorescein (H_2_DCF), which is oxidised by hydrogen peroxide, the hydroxyl radical, and singlet oxygen to form the fluorescent 2′,7′-dichlorofluorescein (DCF) [45,46]. After 1 h of incubation with 0.6 mM allyl alcohol, the cells were centrifuged, washed, and suspended in a 100 mM phosphate buffer, pH 7.0, containing 0.1% glucose and 1 mM EDTA. The kinetics of the increase in fluorescence, due to the oxidation of the fluorogenic probes, was measured using an Infinite 200 microplate reader (Tecan Group Ltd., Männedorf, Switzerland). Measurements were performed for 30 min at 28 °C at λ_ex_ = 518 nm and λ_em_ = 605 nm for DHET, and at λ_ex_ = 495 nm and λ_em_ = 525 nm for H_2_DCF-DA. ROS content was expressed as the rate of fluorescence increase per minute.

### 4.8. Determination of NAD(H) and NADP(H) Content

The NAD(H) and NADP(H) content in the yeast cells was assessed using the NAD^+^/NADH-Glo and NADP^+^/NADPH-Glo Assay kits (Promega, Madison, WI, USA), following the manufacturer’s protocol and own modifications [24]. After 1 h incubation with 0.6 mM allyl alcohol, the cell density (number of cells per mL) was determined using a Malassez chamber. The cells were centrifuged, washed twice with sterile water, and suspended at a density of 2 × 10^6^ cells/mL in a PBS buffer. The cell suspension was then incubated in a 1:1 ratio with lysis solution (0.2 M NaOH containing 1% dodecyltrimethylammonium bromide (DTAB)) for 15 min with intense shaking. After incubation, the samples were split into separate tubes for measuring NAD^+^, NADH, NADP^+^, and NADPH. The subsequent steps of the procedure were performed according to the manufacturer’s protocol. The luminescence signal, which is proportional to the amount of NAD^+^, NADH, NADP^+^, and NADPH, was recorded over 3 h at 25 °C using an Infinite 200 microplate reader (Tecan Group Ltd., Männedorf, Switzerland). The value of the blank was subtracted each time. The results were presented as the content of individual pyridine cofactors and the NADPH/NADP^+^ ratio.

### 4.9. RNA Samples

The RNA samples were obtained using the GeneMATRIX Universal RNA Purification Kit (EURx, Gdansk, Poland), following the manufacturer’s protocol. After 1 h incubation with 0.6 mM allyl alcohol, the cells were centrifuged, washed twice with sterile water, and suspended at a density of 5 × 10^7^ cells/mL in the spheroplast buffer (1 M sorbitol, 0.1 M EDTA, and 0.1% β-mercaptoethanol). The cells were then incubated with lyticase (250 U per sample) for 30 min at 30 °C. The resulting spheroplasts were used for RNA isolation. The RNA samples were stored at −80 °C, and each sample was thawed only once. Four independent biological replicates were prepared for each strain. The concentration and purity of the RNA samples were measured using a NanoQuant Plate on an Infinite 200 microplate reader (Tecan Group Ltd., Männedorf, Switzerland) at a wavelength of 260 nm/280 nm.

### 4.10. Real-Time PCR

A total of 500 ng of RNA, which had been treated with DNase I (Roche, Mannheim, Germany) for 60 min at 25 °C (at a concentration of 10 U per 1 µg of RNA), was used for reverse transcription. The smART First Strand cDNA Synthesis Kit (EURx, Gdansk, Poland) was used to synthesise cDNA following the manufacturer’s protocol, and the samples were stored at −20 °C until use. Real-time PCR was performed using LightCycler 96 (Roche Life Science, Penzberg, Germany) and TaqMan chemistry. Briefly, the cDNA sample was diluted and mixed with FastStart Essential DNA Probes Master (Roche, Mannheim, Germany) and TaqMan Gene Expression Assays (Applied Biosystems, Life Technologies, Pleasanton, CA, USA). Gene expression levels of the *GSH1*, *GLR1*, *TRX1*, *TRX2*, *TRR1*, *ZWF1*, *GND1*, *ALD6*, and *ADH1* genes were tested. The *ACT1* gene was used as an internal control. Relative gene expression was calculated using the −ΔC_T_ method to compare the expression of each gene across all tested strains.

### 4.11. Protein Extraction

After 1 h incubation with 0.6 mM allyl alcohol, the cells were centrifuged, washed twice with sterile water, and suspended in a cold homogenization buffer (20 mM phosphate buffer, pH 6.8, containing 1 mM EDTA, 0.2% DTT, and 1 mM PMSF). The biomass was then disrupted using 0.5 mm glass beads in six cycles of 30 s, with intervals between each cycle for cooling the sample on ice. The sample was subsequently centrifuged at 14,000× *g* for 15 min at 4 °C. The resulting supernatants were transferred to new tubes and immediately frozen at −80 °C. Four independent biological replicates were prepared for each strain. Protein concentration was determined using the Coomassie Protein Assay Reagent (Thermo Scientific, Waltham, MA, USA).

### 4.12. Enzyme Assays

γ-Glutamate-cysteine ligase (γ-GCS) activity was determined following the method of Watanabe et al. [47] with own modification [10]. The rate of ATP usage during the γ-GCS reaction was measured by the reaction coupled with lactic dehydrogenase and pyruvate kinase (PK/LDH enzymes mixture for determination of ADP; P0294 Sigma-Aldrich, Poznan, Poland) by the decrease in NADH absorbance at λ = 340 nm using a Cary 50 spectrophotometer (Varian Ltd., Palo Alto, CA, USA). The coupled reaction was performed at a temperature of 37 °C in a mixture containing 50 mM HEPES buffer, pH 7.0, 12 mM glutamate, 4 mM MgCl_2_, 30 mM ATP, 12.5 mM cysteine, 0.5 mM phosphoenolpyruvate, 0.9 U PK, 1.6 U LDH, 0.1 mM NADH, and the protein extract sample. Appropriate control reactions (without the sample and the PK/LDH mixture) were performed and subtracted. γ-GCS activity was calculated using an extinction coefficient of 6.22 mM^−1^ cm^−1^ and expressed in arbitrary units.

Glutathione reductase (GR) activity was determined by measuring the rate of decrease in NADPH absorbance at λ = 340 nm using a Cary 50 spectrophotometer (Varian Ltd., Palo Alto, CA, USA). The reaction mixture contained 50 mM phosphate buffer, pH 7.0, 0.5 mM DTPA, and 80 µM NADPH. To exclude non-specific NADPH oxidation, the reaction mixture was incubated with the protein extract sample for 1 min before the addition of 2 mM GSSG (final concentration), after which the absorbance was recorded. GR activity was calculated using an extinction coefficient of 6.22 mM^−1^ cm^−1^ and expressed in arbitrary units.

Total PP pathway dehydrogenases activity (the sum of both glucose-6-phosphate dehydrogenase (Zwf1p) and 6-phosphogluconate dehydrogenase (Gnd1p and Gnd2p) activities) and 6-phosphogluconate dehydrogenase activity were determined spectrophotometrically by measuring the rate of NADP^+^ reduction at λ = 340 nm following the method of Tian et al. [48] and own modifications [24]. Zwf1p activity was calculated by subtracting the activity of Gnd1p and Gnd2p from the total enzyme activity. The total dehydrogenase activity was obtained using 0.2 mM NADP^+^, 0.4 mM D-glucose-6-phosphate, and 0.4 mM 6-phosphogluconate as reaction substrates. These were added to a 100 mM Tris-HCl buffer, pH 8.0, containing 1 mM MgCl_2_. To obtain Gnd1p and Gnd2p activity, only 0.2 mM NADP^+^ and 0.4 mM 6-phosphogluconate were used as reaction substrates. The kinetics of absorbance increase were recorded using an Infinite 200 microplate reader (Tecan Group Ltd., Männedorf, Switzerland) at λ = 340 nm. The activity was expressed in arbitrary units.

### 4.13. Statistical Analysis

The results are presented as the mean ± SD from at least three independent experiments. Statistical analysis was performed using the Statistica 13.3 software. The statistical significance of the differences between the WT and pentose phosphate pathway mutant strains was evaluated using one-way ANOVA and Dunnett’s post hoc test. Differences between cells treated with allyl alcohol and untreated control cells were assessed using a *t*-test for independent samples. The values were considered significant at *p* < 0.05.

## 5. Conclusions

The toxicity of acrolein, which is produced within cells from its precursor allyl alcohol, primarily involves the depletion of glutathione, even after short-term exposure, resulting in a redox imbalance. Consequently, cellular response is multidimensional and occurs through attempts to increase glutathione synthesis, changes in the thioredoxin system, and stimulation of NADPH synthesis. The present findings demonstrate that tolerance to acrolein requires coordinated glutathione and NADPH-dependent systems, and that compensatory metabolic rewiring may sometimes paradoxically intensify stress.

## Data Availability

The raw data supporting the conclusions of this article will be made available by the corresponding author upon request.

## Supplementary Materials

The following supporting information can be downloaded at: https://www.mdpi.com/article/10.3390/ijms26189047/s1.
