# Supplementary material for: Disorders of Redox Homeostasis and Its Importance in Acrolein Toxicity
Source: Int J Mol Sci. 2025 Sep 17;26(18):9047. doi: 10.3390/ijms26189047 (PMC12469781; doi:10.3390/ijms26189047)
Supplement: Supplementary file 1 [file ijms-26-09047-s001.zip › ijms-3818240-supplementary.pdf]

Supplementary Material

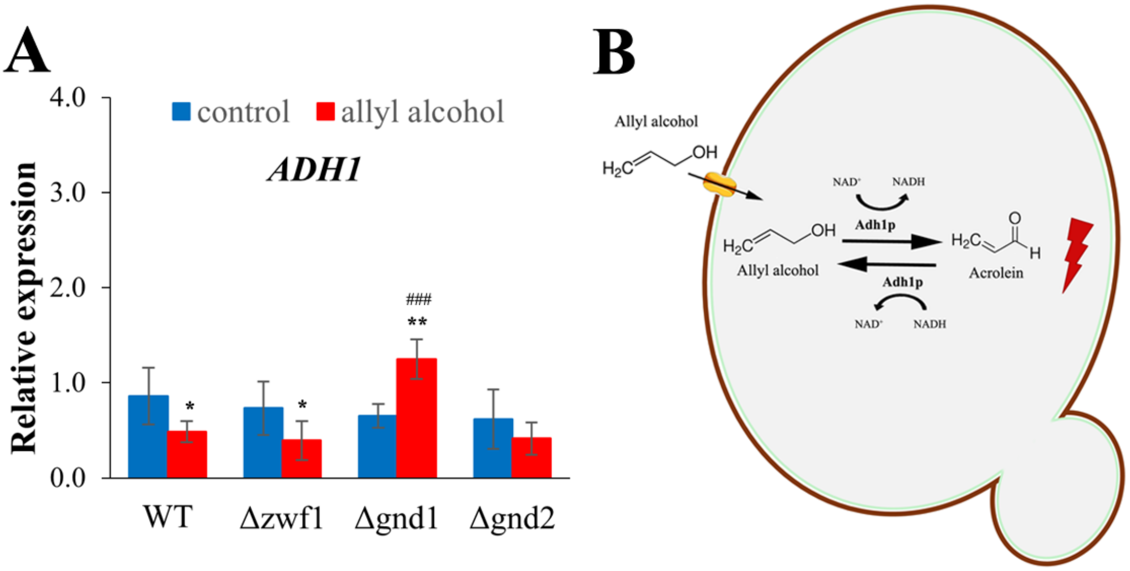

Figure S1. Metabolism of allyl alcohol and expression of alcohol dehydrogenase 1 gene in the WT and pentose phosphate pathway mutant strains after treatment with 0.6 mM allyl alcohol (AA). The relative *ADH1* (**A**) gene expression was determined by qPCR assay with TaqMan probes. The relative gene expression was calculated with the -ΔCT method for comparison of the expression of one gene in all the tested strains. The results are presented as the mean ± SD from at least three independent experiments. The statistical significance of the differences between the values obtained for the WT and mutant strains was evaluated using one-way ANOVA and Dunnett's post hoc test. The differences between the cells treated with allyl alcohol vs. untreated control cells were evaluated using the *t*-test for independent samples. The values were considered significant at a *p*-value < 0.05. Used designations: * $p < 0.05$, ** $p < 0.01$ comparing cells treated with allyl alcohol vs. untreated control cells; ### $p < 0.001$ comparing mutants vs. WT strain. Scheme of allyl alcohol (AA) metabolism and conversion to acrolein (Acr) in the *Saccharomyces cerevisiae* yeast (**B**). Alcohol dehydrogenases (mainly Adh1p) oxidise allyl alcohol to acrolein (Acr)—a highly reactive and toxic aldehyde, and the exposure of the yeast to AA leads to a considerable increase in acrolein content in the cells.

Table S1. The disturbance of redox homeostasis caused by allyl alcohol/acrolein action in strains with disorders in PPP-dependent NADPH production. Comparison: cells treated with 0.6 mM allyl alcohol for 1 h vs. untreated control cells.

**Parameters**	**STRAIN**			
	WT	Δ*zwf1*	Δ*gnd1*	Δ*gnd2*
Cell viability	↓	↓	↓↓	↓
Growth rate and budding ability of cells	↓	↓↓	↓↓↓	↓
Mitochondria activity/morphology	↓↓	↓	↓↓↓	↓
ATP content	↓↓	—/↑	↓↓↓	—/↑
NAD^+ content	—	—	↓	—
ROS content	↑	↑	↑	↑
Reduced (GSH) and total glutathione levels	↓↓↓	↓↓↓	↓↓↓	↓↓↓
Total thiol group content	↓↓	↓↓	↓↓	↓↓
Oxidized glutathione (GSSG) level	↓	↓	—	↓
GSH/GSSG ratio	↓↓↓	↓↓↓	↓↓↓	↓↓↓
Attempts to increase glutathione synthesis	↑	↑	—	↑
Attempts to increase glutathione reduction	↑	↑	—/↑	↑
NADPH content	↑↑↑	↑↑	—	↑
$NADPH/NADP^+$ ratio	↑	—/↓	↓	—
Change of the PPP-dependent NADPH generation	—/↑	—	↓	—/↑
Change of the ALD6-dependent NADPH generation	↑↑	↑	↑↑↑	↑↑↑
Change of the thioredoxin system (*TRX2* and *TRR1* genes)	↑↑	↑↑	↑↑	↑↑

Legend: ↑↑↑ - significant increase; ↑↑ - increase; ↑ - slightly increase; ↓ - slightly decrease; ↓↓ - decrease; ↓↓↓- significant decrease; — no change.
